# Enhancing multiple object tracking performance with noninvasive brain stimulation: a causal role for the anterior intraparietal sulcus

**DOI:** 10.3389/fnsys.2015.00003

**Published:** 2015-02-05

**Authors:** Eric J. Blumberg, Matthew S. Peterson, Raja Parasuraman

**Affiliations:** Arch Lab, Department of Psychology, George Mason UniversityFairfax, VA, USA

**Keywords:** tDCS, brain stimulation, multiple object tracking, anterior intraparietal sulcus, spatial attention

## Abstract

Multiple object tracking (MOT) is a complex task recruiting a distributed network of brain regions. There are also marked individual differences in MOT performance. A positive causal relationship between the anterior intraparietal sulcus (AIPS), an integral region in the MOT attention network and inter-individual variation in MOT performance has not been previously established. The present study used transcranial direct current stimulation (tDCS), a form of non-invasive brain stimulation, in order to examine such a causal link. Active anodal stimulation was applied to the right AIPS and the left dorsolateral prefrontal cortex (DLPFC) (and sham stimulation), an area associated with working memory (but not MOT) while participants completed a MOT task. Stimulation to the right AIPS significantly improved MOT accuracy more than the other two conditions. The results confirm a causal role of the AIPS in the MOT task and illustrate that tDCS has the ability to improve MOT performance.

## Introduction

Multiple object tracking (MOT) is a dynamic, effortful task that assesses how many moving objects a person can attend to over a short period of time (Pylyshyn and Storm, 1988). In the traditional MOT paradigm, participants are presented with multiple objects (e.g., circles) on a monitor and are instructed to track a subset of those objects. The objects move independently and continuously around the screen, and after they stop, participants attempt to indicate which objects they had been tracking. Accuracy, or the proportion of correctly identified targets, is generally used to measure MOT performance.

MOT tasks have been used to evaluate attention capacity (Alvarez and Franconeri, 2007; Horowitz and Cohen, 2010), mechanisms of perceptual organization (Yantis, 1992; Scholl et al., 2001), and distributed attention (Sears and Pylyshyn, 2000). While MOT is a process-intensive task involving attention, object selection, object tracking, memory, and multiple types of eye movements, a number of studies (Pylyshyn and Storm, 1988; Cavanagh and Alvarez, 2005) have illustrated that on average, individuals have a tracking accuracy of 85% for two objects, and as the number of items to track increases, accuracy decreases sharply. In addition, there are marked inter-individual differences in MOT tracking capacity, reflecting inter-individual variation in spatial ability (Oksama and Hyönä, 2004). Given such variability, it is important to understand the underlying neural mechanisms involved in performance of dynamic attentional tasks such as the MOT.

Several researchers (Culham et al., 1998, 2001; Jovicich et al., 2001; Howe et al., 2009) have used functional magnetic resonance imaging (fMRI) to identify the brain areas associated with MOT. Given the number of perceptual and attentional processes involved in MOT, it is not surprising that the fMRI studies have implicated 12 unique brain areas recruited during MOT. Culham et al. (1998) concluded that 11 different brain areas were recruited during MOT whereas Jovicich et al. (2001) identified 12 areas, 9 of which were consistent with the previous work by Culham et al. These studies identified brain regions sensitive to attention, motion, and areas involved in eye movements. However, Howe et al. (2009) identified a number of issues with the previous fMRI and MOT studies, the greatest of which was that the studies did not correctly differentiate brain areas specifically related to tracking objects vs. attending to objects, a critical differentiation in analysis of MOT performance.

After controlling for the effects of attention, Howe et al. (2009) concluded that the frontal eye fields (FEF), anterior intraparietal sulcus (AIPS), the superior parietal lobule (SPL), posterior intraparietal sulcus (PIPS), and the human motion areas (MT+) were all consistently activated during MOT. The FEF and SPL are involved with the generation and execution of eye movements and spatial attention, processes clearly involved with visually tracking objects (Nobre et al., 1997; Donner et al., 2000). Area MT+ plays a critical role in motion-based tasks, and might potentially be responsible for updating location information (d’Avossa et al., 2007). Recent evidence has suggested that the PIPS plays a role in attention to both stationary and moving objects, and may be responsible for managing pointers to the spatial locations of attended objects. PIPS and MT+ may also interact to support MOT with the PIPS involved in attending to the items and MT+ associated with updating of locations (Howe et al., 2009). The AIPS was identified to be active only when objects were moving, suggesting a dissociation between tracking moving objects and attending to stationary ones, and indicating that it plays a crucial role within the identified attention network. In addition, AIPS has been shown to be sensitive to tracking load, with greater activation associated with increased number of items to be tracked (Culham et al., 2001; Jovicich et al., 2001). Supporting this view, a lesion study conducted by Battelli et al. (2001) showed that individuals with a unilateral right parietal lesion were significantly worse at tracking objects in the contralateral field even when only one object was presented in each visual field. Furthermore, Battelli et al. (2009) provided initial evidence supporting the causal role of the AIPS in MOT performance by demonstrating that MOT performance was inhibited by transcranial magnetic stimulation (TMS) over the right and left intraparietal sulcus but not MT+.

A method that can provide evidence for a positive causal relationship between the AIPS and MOT is transcranial direct current stimulation (tDCS). It involves the application of small amounts of constant direct electric current (1–2 mA) with electrodes attached to the scalp. A positive polarity (anode) is typically used to stimulate neuronal function and enhance performance, while a negative polarity (cathode) is used to inhibit neuronal activity. The electric current is thought to affect the resting potential of cortical neurons (Bindman et al., 1964; Antal et al., 2001) and also synaptic efficacy (Rahman et al., 2013), which in turn increases their sensitivity, leading to an increased likelihood of firing while performing a task. (See Bikson et al., 2004, for a deeper explanation on the neural affects of tDCS). The standard current values for active stimulation conditions can fluctuate up to 2 mA while control/sham levels are either 0.1 mA or a 2 mA ramp-up and immediate ramp-down (Clark et al., 2012). No serious side effects have been associated with normal tDCS operations for 30 min or less of prolonged stimulation (Bikson et al., 2009).

Research by Andrews et al. (2011) has indicated that the effects of tDCS are not global, and only occur when administered in a specific manner: the stimulation must be applied so that stimulation targets areas that are involved in the task being trained on. tDCS is thought to facilitate changes in active neurons and pathways, and those pathways must be active while the stimulation is being administered in order to show a benefit. Through the excitatory (anodal) and inhibitory (cathodal) affects on cell membranes, tDCS can improve our understanding of brain function and its corresponding behavioral correlates.

The present study used tDCS to provide a unique approach to investigating the causal role of the AIPS and of evaluating the plasticity of MOT. To demonstrate that the effects of stimulation are focal rather than global in nature both a target and a control site for stimulation were chosen. As discussed previously, the right AIPS was chosen as the targeted experimental site for potential enhancement of MOT performance. The left dorsolateral prefrontal cortex (DLPFC) was chosen as a control stimulation site because previous fMRI studies have shown that it is minimally involved, if at all, in MOT performance (Howe et al., 2009). In contrast, data from Culham et al., 2001 suggests a right lateralized recruitment in frontal brain areas during MOT. Stimulation of this area may lead to inadvertent affects on MOT performance, and because we focused on the right AIPS in the present study, we used the left DLPFC for the control stimulation condition. In addition to the active stimulation control, we also included a sham stimulation of the left DLPFC (to control for a placebo effect). The sham condition is included to control for placebo effects as previous studies have failed to identify meaningful effects of sham stimulation on task performance (Berryhill et al., 2014). Participants were naïve as to the relationship between scalp location and its corresponding behavioral outcomes, making the sham stimulation location unimportant.

We hypothesized that tDCS would improve performance in the right AIPS stimulation condition and that there would be differences in performance between participants stimulated over the right AIPS compared to those stimulated over the left DLPFC in both active and sham conditions. To assess the possible interaction of stimulation with the processing demand associated with attentional tracking, we administered both a low and a high tracking load version of the MOT. We anticipated that an effect of tDCS would be greatest for the high tracking load and may even be absent in the low load condition due to ceiling effects for tasks already at or near ceiling in performance (Ball et al., 2007; Schmiedek et al., 2010; Jaeggi et al., 2011). In addition, previous researchers have suggested that tDCS may be more beneficial for novices/lower performers than for experts/higher performers (Bullard et al., 2011; Tseng et al., 2012; Blumberg et al., 2014; Foroughi et al., 2015) and that tDCS may be more effective in difficult tasks (Berryhill et al., 2014), suggesting that both task difficulty and individual abilities may play a critical role in the effectiveness of stimulation.

## Materials and methods

### Participants

Forty-eight undergraduates participated in the experiment (28 females) with an average age of 19 years (range from 18–32). Participants met the following conditions: (1) right handed; (2) normal or corrected to normal vision; and (3) English as a first language. Participants were randomly assigned to one of the three between-subject stimulation conditions while they performed the MOT task under two tracking loads. Sixteen subjects were assigned to each condition. Participants were given course credit for the their participation. All participants gave written informed consent to participate in a protocol approved by the George Mason Institutional Review Board.

### Task and equipment

#### MOT task

**Figure 1:**
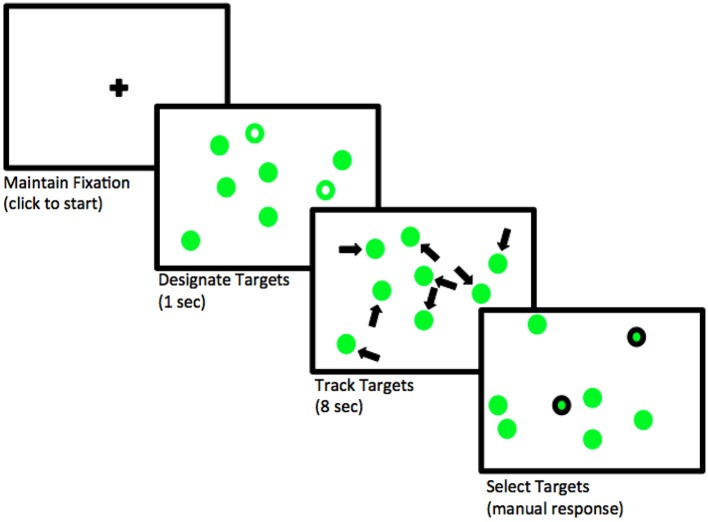
**Sequence of actions within the MOT task**.

Participants engaged in a computer-based MOT task on a Dell 15” inch LCD monitor at a distance of 40 cm from the screen. The experimental stimuli consisted of eight green circles (two or four of the circles were targets). The circles were 1° of visual angle in size. Each trial consisted of three steps. The eight circles initially appeared as static images (no movement) while the target circles flashed for 1 s. Then the circles moved continuously and independently for 8 s, and could overlap as they traveled across the screen. The circles moved at a constant rate of 13°/sec and in constant directions (when they encountered the border of the screen they were redirected in another direction based upon the angle of impact with the border). After the circles stopped, participants selected the target circles with mouse clicks. The experimental sequence can be seen below in Figure 1. Participants tracked two circles (25% of all the circles) in the low load condition and four (50% of all the circles) in the high load condition.

#### tDCS

tDCS was applied using an ActivaDose II Iontophoresis Delivery Unit. Current was constantly supplied to two electrode pads with 11 cm^2^ saline soaked sponges that were attached (with self-adhesive bandage strips) to the participant’s scalp and shoulder. The anode was placed on the scalp while the cathode was placed on the contralateral upper arm, consistent with a non-cephalic montage (Falcone et al., 2012; McKinley et al., 2013). Subjects were randomly assigned to one of three stimulation conditions: AIPS active anodal stimulation, DLPFC active anodal stimulation, and DLPFC sham stimulation. In the AIPS experimental condition the anode was placed near CP4 in the 10–20 EEG system while the cathode was placed on the contralateral upper arm. We used Soterix Medical’s HDExplore software to identify an appropriate montage to best target the AIPS. A standard adult male head was incorporated for the model; therefore, a single current flow model was identified and applied for all participants. We modeled a number of different montages before identifying scalp site CP4 as the site that best activated AIPS. In both the active and sham control conditions the anode was placed near electrode site F3 in the 10–20 EEG system with the cathode placed on the contralateral upper arm (right). F3 is a commonly used site when modulating the DLPFC (Coffman et al., 2014). Participants in both experimental conditions were given 2.0 mA of stimulation for 30 min. Participants in the sham condition received a 2.0 mA ramp-up and immediate ramp-down to 0 mA lasting 30 s. The brief amount of stimulation provided participants with the full sensation of tDCS.

### Design

A 3 × 2 mixed design was employed. The between-subjects variable (stimulation site) had the following levels: AIPS active, DLPFC active, and DLPFC sham. The within-subjects variable was tracking load (low or high). Each participant completed six blocks of 44 trials (three blocks during baseline testing and three blocks while stimulation was administered). The trials in each block were randomized with an equal representation of low and high tracking trials.

### Procedure

Upon arrival participants were asked to read and sign the informed consent form outlining the nature of the task and any risks/benefits they may receive for participating. The Snellen near-sightedness exam was administered to test vision (20–30 or better vision required). Participants were then instructed on how to perform the MOT task. Participants completed a baseline of three blocks of 44 trials to test their baseline performance. The three blocks were completed back-to-back without any breaks.

Following baseline testing, the experimenter prepared the tDCS setup. Participants were given the DCS Sensation Questionnaire (Scheldrup et al., 2014) at three time points throughout the stimulation subsession (approximately 1-, 10-, and 30 min post stimulation onset) measuring how much itching, heat/burning, and tingling the participant felt at that moment. Immediately following the first administration of the sensation questionnaire participants completed a demographic and video game questionnaire. After they finished the questionnaires they completed the second sensation questionnaire. Participants then completed the final three blocks of the MOT task (132 total trials) while tDCS was being administered. After completing the trials the tDCS unit was turned off and the electrodes were removed. Finally, each participant completed the third sensation questionnaire and then was debriefed about the experiment.

## Results

### MOT accuracy

**Figure 2:**
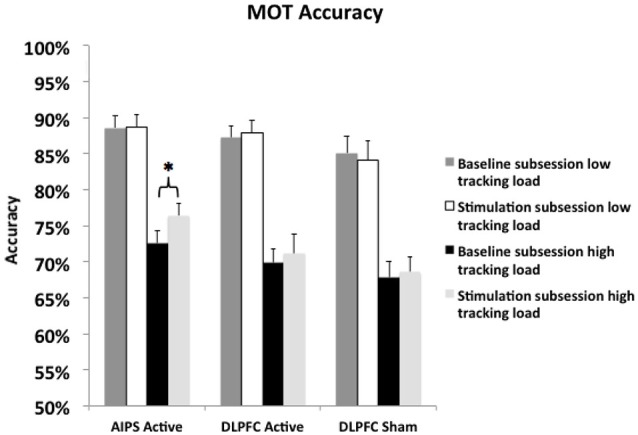
**MOT accuracy ± SEM (standard error of the mean) across stimulation sites broken down by time point (baseline and stimulation) and tracking load (low and high)**. * indicates significant difference.

The primary goal of this experiment was to investigate whether a relatively short period of brain stimulation (30 min) could be used to improve MOT performance, thereby establishing a positive causal role of the right AIPS in MOT performance. The behavioral and dependent variable in the experiment was MOT accuracy. Accuracy was calculated by dividing the number of correctly identified targets by the total possible targets for each trial. Accuracy scores were then created for both the baseline and stimulation subsessions by averaging the accuracy scores across trials and blocks within each subsession. Separate accuracy scores were created for both load conditions leaving each participant with four different accuracy scores (see Figure 2).

### Baseline comparison

We initially tested whether baseline performance across stimulation conditions was significantly different from one another. Two separate (low and high tracking loads) one-way analysis of variance (ANOVA) were conducted because we did not want a potential ceiling effect in the low tracking load condition (hypothesized* a priori*) to reduce the likelihood of finding an effect in the high tracking load condition. The ANOVA for the low tracking load condition failed to identify a significant main effect of stimulation condition (*F*_(2,45)_ = 0.87, *p* > 0.20). The ANOVA for the high tracking load condition also did not reveal a significant main effect of stimulation condition (*F*_(2,45)_ = 1.34, *p* > 0.20) suggesting that baseline performance was not significantly different across stimulation groups in either tracking load condition.

### Condition specific stimulation effect

We then conducted a 2 × 2 × 3 mixed-design ANOVA with subsession and tracking load as the within-subjects factors, stimulation site as the between-subjects factor, and MOT accuracy as the independent variable. A self-assessment of first person shooter video game experience was initially included in the analysis as a covariate because prior research (Green and Bavelier, 2006) suggested it predicts MOT performance, however, this effect was not significant in the present study and was therefore removed from the subsequent analyses. The analysis revealed a 2-way interaction between tracking load and subsession (*F*_(1,45)_ = 5.24, *p* < 0.05, $\eta_{partial}^{2}$ = 0.10), a main effect for subsession (*F*_(1,45)_ = 351.14, *p* < 0.01), and a main effect for tracking load (*F*_(1,45)_ = 4.34, *p* < 0.05). Tests of simple main effects for the two-way interaction using a Bonferroni correction (*α* = 0.05) revealed that within the high tracking load condition, MOT performance was significantly greater in the stimulation subsession compared to baseline, (*F*_(1,45)_ = 9.5, *p* < 0.01, $\eta_{partial}^{2}$ = 0.17). The three-way interaction was not significant (*p* > 0.10).

To better test our initial hypothesis about whether tDCS stimulation applied to the AIPS can improve MOT performance, a series of planned paired samples *t*-tests were conducted to identify if stimulation improved MOT accuracy beyond that of baseline. Six, separate paired samples *t*-test using a Ŝidák correction (*α* = 0.0063, given six related tests) were conducted comparing each baseline score to its corresponding stimulation score (low and high tracking load for each stimulation site). A significant difference in performance was identified in the high tracking load condition between AIPS baseline (*M* = 76.42%, *SE* = 1.66) and AIPS stimulation (*M* = 72.54%, *SE* = 1.86), *t*_(15)_ = 4.10, *p* = 0.00047, *d* = 1.03 illustrating a 4% improvement in MOT accuracy, see Figure 2. No other *t*-test reached significance (largest *t* = 1.0; smallest *p* = 0.33). Given that stimulation did not affect performance in the low tracking load condition across any stimulation condition and we did not make any *a priori* predictions, the low tracking load condition was excluded from the following analyses.

### Comparison of stimulation effects across conditions

In addition to testing for changes in performance due to stimulation, we also examined whether stimulation led to group differences. Given the *a priori* hypothesis that in the high tracking load condition the AIPS stimulation condition would be significantly different from the two DLPFC control conditions, the DLPFC active (*M* = 71.16%, *SE* = 2.67) and DLPFC sham (*M* = 68.56%, *SE* = 2.12) groups were initially compared against one another to identify any differences. An independent samples *t*-test using a Ŝidák correction (*α* = 0.025, given two related tests) was conducted to compare performance across the two control conditions in the stimulation subsession. The analysis did not reveal a significant difference between the two control groups, *t*_(30)_ = 0.45, *p* = 0.41. The two DLPFC control conditions were therefore collapsed into one control condition in the subsequent analysis, leaving two levels of the stimulation variable (AIPS active and DLPFC control).

**Figure 3:**
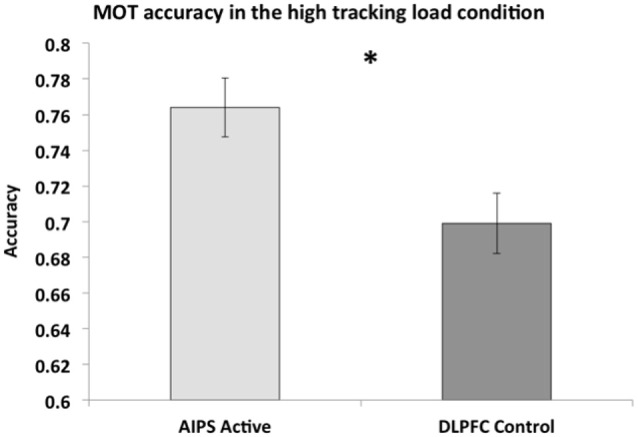
**MOT accuracy in the high tracking load condition + SEM (standard error of the mean)**. * indicates significant difference.

We then tested if performance in the AIPS active and DLPFC control condition were significantly different from one another in the stimulation subsession, examining if AIPS stimulation led to better MOT performance compared to the DLPFC control. An independent samples *t*-test using a Ŝidák correction (*α* = 0.025) was conducted to compare performance between the AIPS active (*M* = 76.42%, *SE* = 1.67) and DLPFC control (*M* = 69.86%, *SE* = 1.69) conditions. The analysis revealed a significant difference *t*_(46)_ = 2.45, *p* = 0.009, *d* = 0.80. The data suggests that in the high tracking load condition, right AIPS stimulation improved MOT accuracy significantly more than in the combined control condition, see Figures 2, 3.

### MOT capacity

Due to the high accuracy scores in the low tracking load condition, an important question becomes: is accuracy a sensitive enough measure to detect performance changes close to ceiling? To answer this question, we non-linearly transformed the accuracy scores into capacity measures (k) according to Horowitz et al. (2007) and Scholl et al. (2001). The capacity measure did not lead to any significantly different outcomes compared to the accuracy measure, therefore, the analyses will not be included in this manuscript, see Table 1 for means.

**Table 1:** **Means for both accuracy and capacity measure across stimulation condition, tracking load, and subsession**.

		Low		High	
		Baseline	Stimulation	Baseline	Stimulation
Accuracy	AIPS Active	88.59	88.64	72.54	76.42
DLPFC Active		87.26	87.97	69.89	71.16
DLPFC Sham		85.09	84.14	67.80	68.56
Capacity	AIPS Active	1.77	1.77	2.88	3.04
DLPFC Active		1.74	1.76	2.77	2.82
DLPFC Sham		1.70	1.68	2.68	2.72

### Baseline vs. change in MOT performance

**Figure 4:**
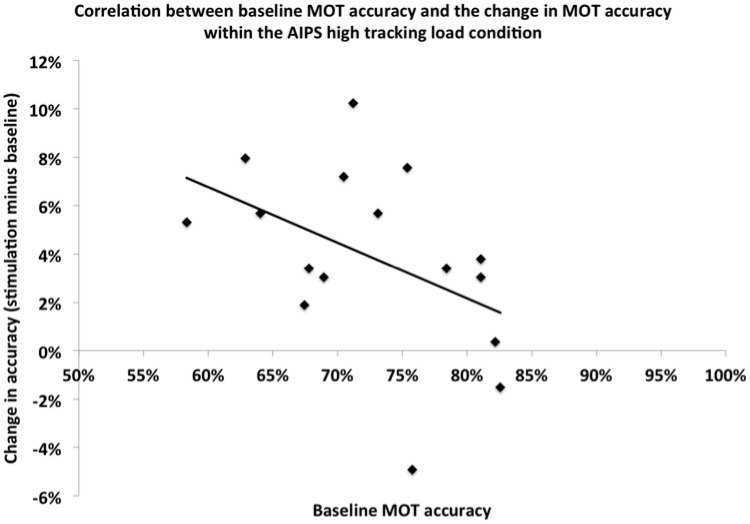
**Correlation between baseline MOT accuracy and the change in MOT accuracy within the AIPS high tracking load condition**.

We also examined whether baseline MOT accuracy predicted the amount of improvement exhibited in the right AIPS stimulation condition (high tracking load trials). To accomplish this we compared participants’ baseline MOT accuracy to their change in MOT accuracy (stimulation minus baseline). The two scores were negatively correlated, *r*_(16)_ = −0.45, *p* < 0.05, see Figure 4. This significant association suggests that tDCS may be more beneficial to individuals with lower baseline MOT abilities. Note also, that all but two of the participants, irrespective of their baseline performance, showed improvement in MOT accuracy with tDCS.

### Rate of MOT improvement

**Figure 5:**
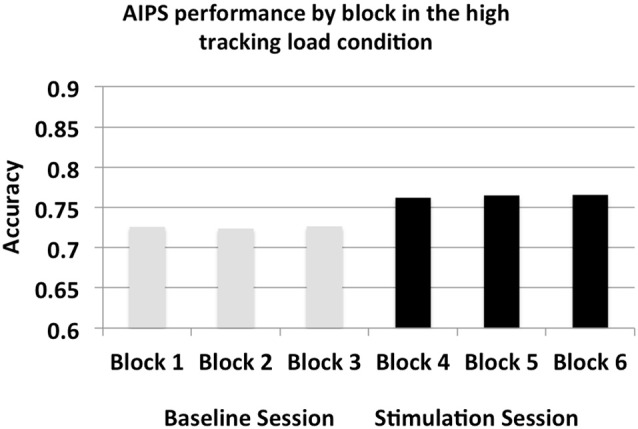
**Accuracy per block in the AIPS stimulation high tracking load condition, subsession differentiated by bar color**.

Additionally, we examined the rate at which stimulation impacted MOT performance in the AIPS stimulation high tracking load condition. We ran a repeated measure ANOVA with block (only in the stimulation subsession) as the within-subjects factor. Block was not significant (*F*_(2,30)_ = 0.04, *p* > 0. 10) indicating that stimulation led to an immediate boost in MOT performance that was sustained across the three blocks (see Figure 5—Blocks 4,5,6).

## Discussion

The present study evaluated the efficacy of using targeted non-invasive brain stimulation to improve understanding of the causal role of the right AIPS in MOT performance through improved learning and skill acquisition of MOT. tDCS was targeted to the right AIPS, a brain area that plays a unique and integral role for tracking multiple objects (Howe et al., 2009). Active anodal stimulation to the right AIPS improved MOT performance in the high tracking load condition but not in the low tracking load condition. Active and sham stimulation of the left DLPFC had no effect in either tracking load condition. This finding suggests that: (1) the right AIPS plays an active role in MOT; (2) modulation of this area by tDCS directly leads to changes in MOT performance; and (3) the effects of the tDCS were focal in nature and not a global enhancement due to stimulation of the entire cortex.

Right AIPS stimulation improved performance in the more difficult tracking load condition where participants’ accuracy was relatively low (~70%) whereas stimulation did not affect performance in the easier tracking load condition (85–90%) where participants were performing at or close to ceiling. These results are consistent with the *a priori* hypotheses and are in line with the previous literature that suggests that tDCS is more beneficial to novices (Bullard et al., 2011) and lower performers (Tseng et al., 2012; Blumberg et al., 2014; Foroughi et al., 2015) and may be more effective when paired with difficult tasks (Jones and Berryhill, 2012; Berryhill et al., 2014). Additionally, cognitive training is not beneficial for individuals already performing at ceiling (Ball et al., 2007; Schmiedek et al., 2010; Jaeggi et al., 2011).

We also identified that in the high tracking load condition, the amount of improvement in MOT was negatively correlated with a participant’s baseline MOT ability. On average, individuals with lower baseline MOT accuracies exhibited greater increases in accuracy compared to those with higher baseline abilities. Tseng et al. (2012) found a similar inverse effect in change-detection ability stimulating a posterior parietal location.

Additionally, we identified that stimulation had an immediate effect on MOT performance in the right AIPS high tracking load condition. Performances across the three blocks in the stimulation subsession were similar, illustrating a constant positive impact of stimulation. We believe this is due to the fact that MOT is a very simple task with little to no learning curve, therefore, stimulation immediately modulated the relationship between the right AIPS and MOT performance.

While research (Green and Bavelier, 2006; Boot et al., 2008) has previously shown that specific types of training such as playing action video games can improve MOT performance, this is the first study to show that brain stimulation can do so too, but in a much shorter time. If used as a tool for accelerated training, tDCS may offer a number of benefits compared to traditional training paradigms. Video game training can take extended periods of time (Green and Bavelier, 2006; Feng et al., 2007; Basak et al., 2008), whereas in this study, tDCS immediately improved spatial tracking performance. Also, specific subsets of the population cannot or do not enjoy playing video games because the games can be difficult to learn, can cause frustration, and can require fine motor control. On the other hand, tDCS requires little or no additional effort from the user apart from the task being performed, making it ideally suited to a larger segment of the population. tDCS focused on the right AIPS, a brain area integral to the attention network (Howe et al., 2009) immediately improved MOT performance, this transient improvement was accomplished in a substantially shorter amount of time than through traditional training programs, however, the effects may not be as significant or as long lasting. While this study provides initial evidence that tDCS can rapidly improve MOT performance, further research should identify if these effects are transferable to other spatial tracking tasks in both basic and complex settings. Brain stimulation over the right AIPS offers a unique method to better understand the function of this area as it relates to MOT. This study adds to the existing literature that the right AIPS plays an active role in MOT and that the neural substrates recruited for MOT exhibit significant plasticity.

Our findings mirror previous tDCS studies that have found effects on perception, attention, and memory abilities (Coffman et al., 2014). For example, Clark et al. (2012) showed increased perceptual learning when tDCS electrodes were targeted to brain areas related to perceptual learning, with the benefit of tDCS being retained for at least 24 h (Falcone et al., 2012). Of particular note is the study by Moos et al. (2012), in which they observed that cathodal stimulation over the right AIPS increased top-down attentional selection. While they applied cathodal stimulation to the same area we did, the two studies measured different aspects of attentional selection. Additionally, TMS has previously been applied to support a causal mechanism between modulation of the IPS and MOT (Battelli et al., 2009). They inhibited IPS function, leading to decreased MOT accuracy. However, our study is the first to illustrate that the causal mechanism is in the positive direction as well. We used tDCS to illustrate the facilitative effects of AIPS stimulation on MOT ability, finding increased MOT accuracy. tDCS is also less invasive and more easily applied making it a more practical tool to accelerate MOT abilities.

This study had some limitations. Although current modeling was used to identify the electrode montage that would best lead to stimulation of AIPS, such modeling involves a number of assumptions that may not always be met, and modeling must be considered as a hypothesis to be tested rather than definitive. That the empirical evidence confirmed the hypothesis and showed that other stimulation sites did not lead to improvement in MOT performance is consistent with the predicted results. Furthermore, we did not directly measure cortical activation in AIPS or other parietal regions as a result of tDCS. However, previous research has illustrated that tDCS does affect neuronal firing (Radman et al., 2009). Additionally, the tDCS electrode montage used in the experiment may have resulted in stimulation of the posterior parietal cortex in general.

Another possible concern involves the baseline performance of the different stimulation/sham groups. While statistically significant differences between groups in MOT baseline performance were not found, one could argue that individuals in the AIPS condition were somewhat better to begin with, so that the difference identified in the stimulation subsession could just reflect these initial differences in performance and random variation or potentially a small tDCS effect. However, it would be highly unlikely that random noise would increase performance in individuals that were already performing at high levels and not for individuals performing poorly. Additionally, it is unlikely given that individuals with the lowest baseline MOT abilities saw the largest increases in MOT performance. If baseline averages were identical (decreasing initial AIPS accuracy) potentially more individuals would see greater benefits in their MOT performance. Task difficulty can also modulate the beneficial effects of tDCS (Jones and Berryhill, 2012; Berryhill et al., 2014). While AIPS stimulation significantly improved MOT accuracy in the four ball tracking condition (an amount at or close to our attention capacity) and not the two ball tracking condition, an even more difficult MOT condition (i.e., tracking six objects) may have resulted in larger effects. Therefore, the findings in this paper may be underestimating the beneficial effect of tDCS on MOT performance. Future research should investigate the limitations of applying tDCS to the intraparietal sulcus, how cathodal stimulation may affect spatial tracking performance, and how tDCS could be used in conjunction with other training programs to improve spatial tracking beyond that of just video game play or tDCS alone. Additionally, it will be very important to identify how long the tDCS effects last, especially if tDCS is used for long-term enhancement of spatial abilities.

The current study is the first to illustrate that brain stimulation can improve MOT accuracy. Stimulation to the AIPS, a central location in the attention network improved MOT accuracy while stimulation to the DLPFC did not. Accelerated training techniques like tDCS can be used to improve perceptual, attention, and memory training programs and to identify the causal relationships between brain and behavior.

## Conflict of interest statement

The authors declare that the research was conducted in the absence of any commercial or financial relationships that could be construed as a potential conflict of interest.
